# Broad‐range necrophytophagy in the flagellate *Orciraptor agilis* (Viridiraptoridae, Cercozoa) and the underappreciated role of scavenging among protists

**DOI:** 10.1111/jeu.13065

**Published:** 2024-11-03

**Authors:** Jannika Moye, Sebastian Hess

**Affiliations:** ^1^ Division for Biology of Algae and Protozoa, Department of Biology Technical University of Darmstadt Darmstadt Germany

**Keywords:** algae, decomposition, flagellates, necrophagy, Rhizaria, scavenger

## Abstract

Protists show diverse lifestyles and fulfill important ecological roles as primary producers, predators, symbionts, and parasites. The degradation of dead microbial biomass, instead, is mainly attributed to bacteria and fungi, while necrophagy by protists remains poorly recognized. Here, we assessed the food range specificity and feeding behavior of the algivorous flagellate *Orciraptor agilis* (Viridiraptoridae, Cercozoa) with a large‐scale feeding experiment. We demonstrate that this species is a broad‐range necrophage, which feeds on a variety of eukaryotic and prokaryotic algae, but fails to grow on the tested fungi. Furthermore, our microscopic observations reveal an unexpected flexibility of *O. agilis* in handling food items of different structures and biochemistry, demonstrating that sophisticated feeding strategies in protists do not necessarily indicate narrow food ranges.

## INTRODUCTION

Protists fulfill diverse important roles in aquatic and soil food webs. While phototrophic representatives, usually referred to as algae, fix carbon dioxide and act as primary producers (Field et al., 1998), heterotrophic forms consume prokaryotes and microeukaryotes by phagotrophy (Sherr & Sherr, 2016) or engage in various mutualistic, parasitic and parasitoid relationships (Garcés et al., 2013; Kim et al., 2008, 2014; Seyedsayamdost et al., 2011). Furthermore, the indirect impact of protists on higher plants, as part of their “microbiome”, was shown as well (Clarholm, 1985; Sapp et al., 2018). All these processes concern the living organisms, as do most protist‐focused studies and ecological concepts (Geisen et al., 2016; Weitere et al., 2018).

The degradation and mineralization of dead biomass is another important ecological process, which is mostly attributed to saprotrophic bacteria and fungi (López‐Mondéjar et al., 2018). These decomposers are the key players in the breakdown of dead biomass by the secretion of enzymes and the uptake of the released nutrients by osmotrophy. On the macroscopic level, detritivores and necrophages, mainly stemming from the animal kingdom, consume dead plant matter and animal tissue, and thereby take part in the destruction of dead biomass in the environment (Hobbie & Villóeger, 2015; Megías et al., 2011). The distinction between necrophages and detritivores is sometimes not very clear, and the terms might be used interchangeably in some cases. However, while detritivores (e.g. lumbricid earthworms) may feed on dead organisms, many necrophages (e.g. sarcophagid flies) do not consume detritus (= partially degraded material from plants, animals, and microbes often mixed with feces and potentially mineral particles).

In protists, necrophagous behavior is still relatively poorly recognized (Potapov et al., 2022; Weitere et al., 2018). This might be due to the fact that the cultivation of algae and protists is usually based on defined culturing media, bacterial growth, or specific prey organisms provided in the live condition (Andersen, 2005; Day et al., 2007; Kirby, 1950). However, there are some exceptions such as the relatively well‐studied thraustochytrids (class Labyrinthulea in the stramenopiles). These saprotrophic protists form large ectoplasmic nets and decompose various substrates, including dead plant and animal tissue, by the secretion of various lytic enzymes (Bongiorni et al., 2005; Iwata & Honda, 2018; Perkins, 1973). They are supposed to play a relevant role as decomposers of plant biomass in marine and estuarine ecosystems (Raghukumar, 2002; Raghukumar et al., 1995; Sathe‐Pathak et al., 1993; Ueda et al., 2015). Other examples include histophagous ciliates (e.g. from the genera *Coleps*, *Paraspathidium*, *Plagiopogon*), which can feed on dead animal tissue (Auer et al., 2004; Bark, 1985; Fenchel, 1969), and the testate amoeba *Netzelia tuberspinifera* (formerly assigned to the genus *Difflugia*; Amoebozoa) scavenging on dead copepods and cladocerans (Han et al., 2011). Finally, there are some reports of nucleariid amoebae (Opisthokonta) consuming the contents of damaged cells of the freshwater alga *Spirogyra* (Zygnematophyceae), while not being able to penetrate healthy cells (Cann, 1986; Cienkowski, 1865; Patterson, 1983). However, all these organisms also feed on live prey and are thus facultative necrophages.

About 10 years ago, a group of algivorous freshwater flagellates, the Viridiraptoridae (Cercozoa, Rhizaria), were discovered (Hess & Melkonian, 2013). These flagellates feed specifically on the cell contents of zygnematophycean green algae, which involves the local and well‐defined perforation of the algal cell wall. The feeding process appears to be highly specialized as it involves cytoskeletal remodeling, i.e. the formation of an F‐actin‐rich lysopodium (Busch & Hess, 2017), and the secretion of carbohydrate active enzymes (Gerbracht et al., 2022; Moye et al., 2022). Both known species, *Viridiraptor invadens* and *Orciraptor agilis*, also accept dead algal cells. In fact, the latter species was described as a bona fide necrophage based on observations in natural samples and a limited feeding experiment (Hess & Melkonian, 2013). In the natural habitat, *O. agilis* was found extracting the dead cells of a small‐celled *Mougeotia* species (Zygnematophyceae). However, subsequent observations of *Orciraptor*‐like flagellates in other natural samples indicate that viridiraptorids could act on a much larger spectrum of potential food/prey organisms.

To test the hypothesis of broad‐range necrophagy in viridiraptorids, we assessed the food range specificity and feeding behavior of *O. agilis* with a large‐scale feeding experiment. Algae, fungi and cyanobacteria were offered in the live and dead condition, and the reactions of *O. agilis* were studied by conventional light microscopy and time lapse photography. Overall, our data provide a much more complete picture of the feeding ecology of this necrophagous flagellate and reveal the astounding flexibility of this protist to handle food of different structure and biochemistry.

## MATERIALS AND METHODS

### Organisms and cultivation

The axenic strains of *O. agilis*, OrcA01 and OrcA03, were grown with freeze‐killed *Mougeotia* sp. (strain CCAC 3626) as previously described (Hess & Melkonian, 2013). *Mougeotia* sp. was cultivated in liquid Waris‐H medium (McFadden & Melkonian, 1986), supplemented with 1% (v/v) bacterial standard medium (0.8% peptone, 0.1% glucose, 0.1% meat extract, 0.1% yeast extract in distilled water (w/v); Melkonian & Weber, 1975) at 15°C under white LEDs (photon fluence rate 10–30 μmol m^−2^ s^−1^, 14:10 h light–dark cycle). The other algal strains used for the feeding experiments were grown in pure Waris‐H under the same light and temperature conditions. The algal strains are available through the Central Collection of Algal Cultures (CCAC) at the University of Duisburg‐Essen (https://www.uni‐due.de/biology/ccac/index.php), the Culture Collection of Algae (SAG) at the University of Göttingen (https://uni‐goettingen.de/de/184982.html), or through the corresponding author upon request. The fungal strains of the Saccharomycetes and Tremellomycetes were grown in yeast extract peptone dextrose medium (YPD; 10 g*L^−1^ yeast extract, 20 g*L^−1^ peptone and 20 g*L^−1^ glucose), while the Leotiomycetes were grown in malt yeast peptone medium (MYP; 7 g*L^−1^ malt extract, 1 g*L^−1^ peptone and 0.5 g*L^−1^ yeast extract; Langer, 1994). All fugal strains were cultivated at room temperature. Leotiomycetes cultures are available through the German Collection of Microorganisms and Cell Cultures (https://www.dsmz.de/), yeast cultures through the corresponding author upon request.

### Feeding experiments

The feeding experiments (in triplicates) were done with live and dead organisms suspended in sterile demineralized water at room temperature (~20°C). Well grown algal and fungal material was freeze‐killed at −26°C, except *Mesotaenium caldariorum* (SAG 648‐1) and *Serritaenia testaceovaginata* (GSM_5G‐4‐thin), which were frozen at −80°C. After thawing, the cells of freeze‐killed cultures were washed with sterile, demineralized water, and then added to densely grown cultures of *O. agilis* in the foraging stage. Similarly, live cells were washed and added to *Orciraptor* cultures. The resulting test cultures were observed in regular intervals with a Motic AE2000 inverted microscope (Motic Hong Kong Limited, Hong Kong). The same microscope equipped with a MikroLive 6.4 MP CMOS camera (MikroLive, Oppenau) was used for time‐lapse photography on selected cultures. High resolution images were taken with a ZEISS Axio Observer 7 inverted microscope with the Plan‐Neofluar 100×/1.3 objective lens, DIC optics, and the Axiocam 512 color (Carl Zeiss, Oberkochen, Germany). The test cultures were categorized depending on the interactions of *O. agilis* with cells, observed perforations of cell walls, feeding success, and growth of the *O. agilis* population (details see results). The experimental cultures were kept until the prey was entirely consumed or *O. agilis* starved to death. In unclear cases the cultures were observed at least 1 month.

### Fluorescence and scanning electron microscopy

Emptied colonies of *Eudorina elegans* (Chlamydomonadales, Chlorophyceae) were stained with a fluorescent wheat germ agglutinin (WGA, Invitrogen) conjugated with TRITC. For this staining the emptied colonies were centrifuged at 1000 *g* for 10 min. The supernatant was subsequently removed, and the colonies transferred onto poly‐L‐lysin (Sigma‐Aldrich) coated coverslips. After settling of 20 min the coverslip was washed with distilled water and then incubated with 5 μg mL^−1^ WGA‐TRITC for 10 min in the dark. The coverslips were washed three times with distilled water and mounted with SlowFade™ Antifade Mountant (Invitrogen) on microscopy slides and sealed with nail polish. The samples were visualized using the ZEISS Axio Observer 7 with the ZEISS Colibri 5 LED illumination system (RGB‐UV) and the filter set 43 HE Cy3 (excitation 550/25, emission 605/70). Resulting Z‐stacks were processed with the software Fiji (Schindelin et al., 2012). Emptied zygnematophytes were prepared for scanning electron microscopy (SEM) as previously described (Moye et al., 2022). In short, the algal cells were collected by centrifugation (1500 *g*, 10 min), mounted on poly‐L‐lysin‐coated coverslips (Sigma‐Aldrich, Darmstadt, Germany), dehydrated in a series of ethanol dilutions (50%, 96%, 100%, 5 min each step), passed through hexamethyldisilazane (Carl Roth, Karlsruhe, Germany), and air‐dried. The cells were then sputter‐coated with gold and imaged with a Zeiss Neon 40 SEM (secondary electron detector, 2.5 kV acceleration voltage; Carl Zeiss, Oberkochen, Germany) or a LEO 430 (LEO Electron Microscopy LTD, Cambridge, Great Britain).

## RESULTS

**FIGURE 1:**
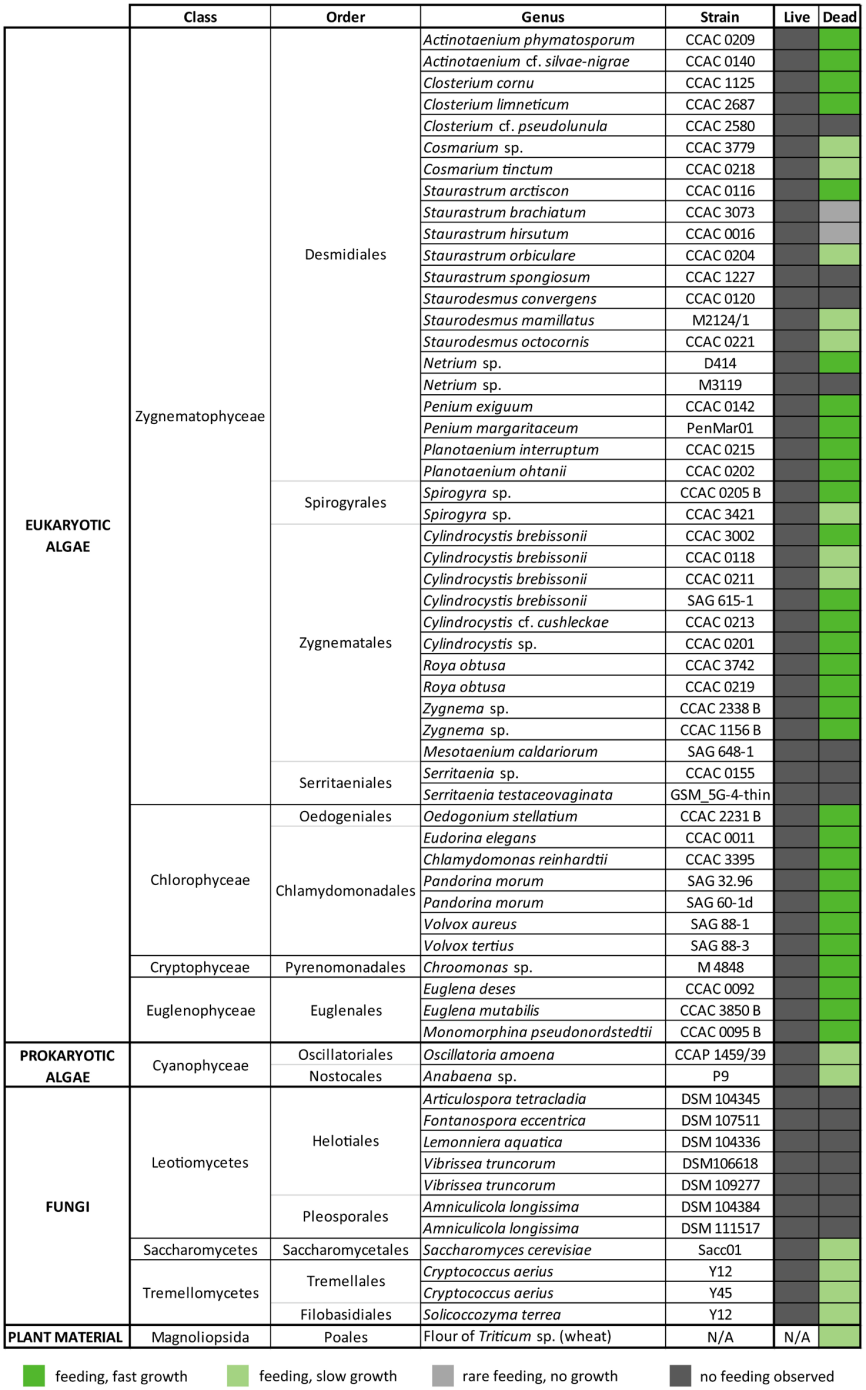
Results of the feeding experiments with *O. agilis*. Tested organisms are listed according to their taxonomic treatment. “fast growth” indicates the impression of a rapid increase in cell number as observed with the natural food alga (Hess & Melkonian, 2013). CCAC = Central Collection of Algal Cultures; DSM = German Collection of Microorganisms and Cell Cultures; SAG = Culture Collection of Algae at Göttingen University.

We tested two strains of *O. agilis* (OrcA01 and OrcA03), which are genetically very close as assessed by their SSU rRNA gene sequences (Hess & Melkonian, 2013). As the strains did not show any marked behavioral differences throughout this study, we only refer to *O. agilis* as a species in the following. Overall, *O. agilis* interacted with the majority of the offered food organisms, which represented a broad phylogenetic and morphological range. The duration of the feeding act differed greatly depending on the attacked alga, ranging from a few minutes (e.g. *Chroomonas* sp.) to several hours (e.g. *Cylindrocystis brebissonii*). This likely relates to the cell wall characteristics of the algae. However, feeding and growth was exclusively observed on freeze‐killed cells, while live algae and fungi were not attacked (Figure 1). In the latter cases, *O. agilis* starved to death within a few days.

### Coccal and filamentous algae (Zygnematophyceae)

**FIGURE 2:**
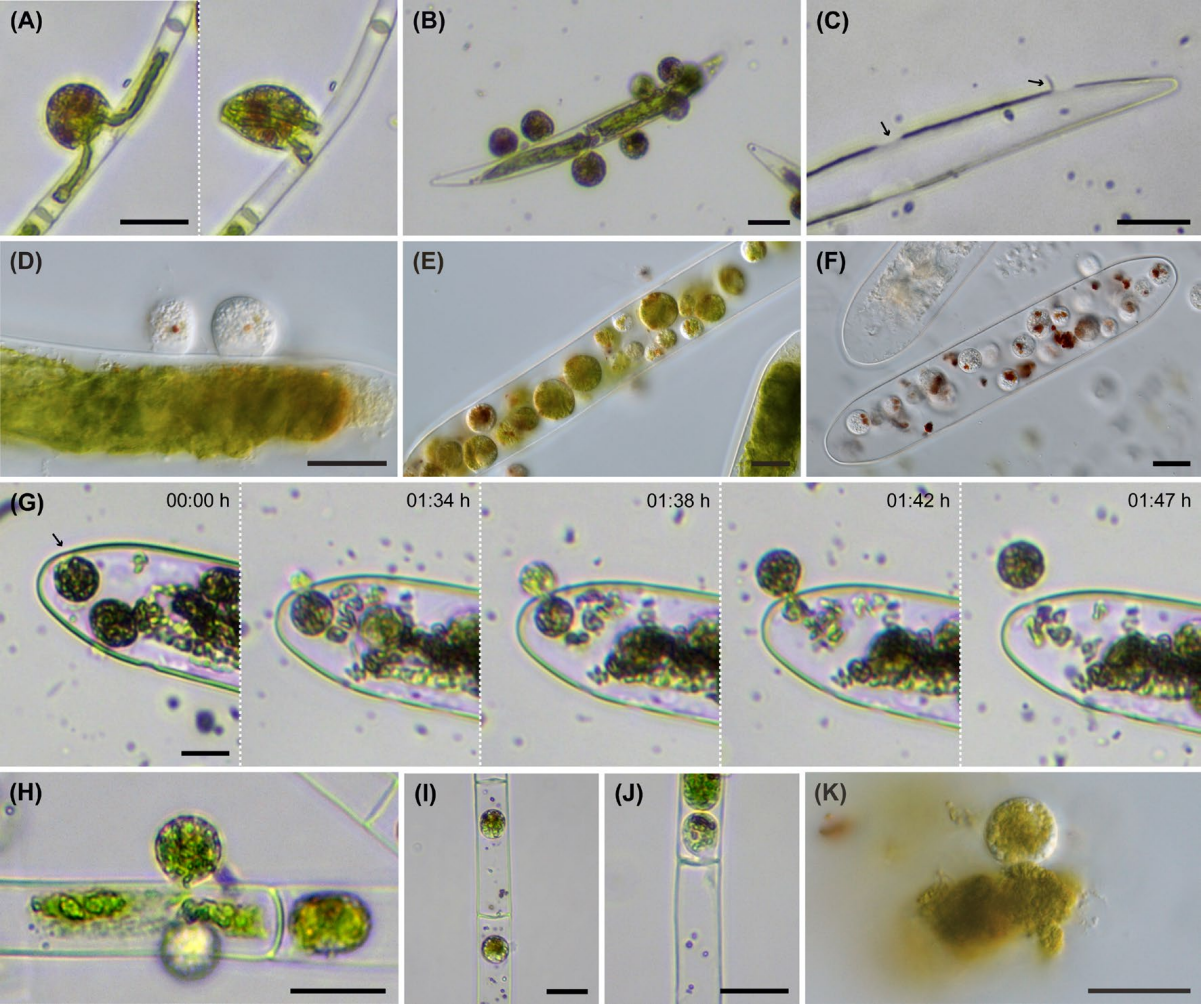
*Orciraptor agilis* (strain OrcA03) feeding on different zygnematophytes. (A) Cell extracting the contents of a small‐celled *Mougeotia* species. (B) Multiple cells attacking *Closterium cornu*. (C) Perforations (arrows) left by *O. agilis* in the cell wall of *Closterium cornu*. Note the attached cell wall disc. (D) Two cells attacking *Penium margaritaceum* with discernible lysopodia. (E) Multiple well‐fed *Orciraptor* cells inside *Penium margaritaceum*. (F) Colorless cells with food remnants inside *Penium margaritaceum*. (G) Time series of *O. agilis* perforating the cell wall of *Penium margaritaceum* from the inside and exiting the algal cell. (H) *Orciraptor* extracting cell contents from a *Spirogyra* sp. filament. (I) Two cells inside a *Spirogyra* sp. filament. (J) Cell penetrating the cross wall of *Spirogyra* sp. (K) Cell feeding on free protoplast material of *Penium margaritaceum*. Scale bars: 20 μm.

*Orciraptor agilis* fed and grew on most of the tested zygnematophytes, including unicellular and filamentous species. The flagellates perforated the algal cell walls as already described for a small‐celled *Mougeotia* species, one of the natural food algae (Hess & Melkonian, 2013), and the desmid *Actinotaenium* cf. *silvae‐nigrae* (Moye et al., 2022). The cells of small‐celled algae are typically emptied by a single *Orciraptor* cell (Figure 2A). Large‐celled zygnematophytes, e.g. *Closterium cornu*, were often attacked by multiple *Orciraptor* cells at the same time (Figure 2B), resulting in multiple perforations (Figure 2C). In many zygnematophyte species, we observed excised cell wall discs still attached after feeding, yet with varying frequency. This aligns well with the observation that *Orciraptor* formed the typical ring‐like lysopodia, which are involved in the enzymatic dissolution of the cell wall (Busch & Hess, 2017; Moye et al., 2022), in all observed instances (Figure 2D). The time required for perforation of the algal wall differed greatly between the zygnematophyte species, up to several hours. The actual food uptake happened either by protoplast extraction or after infiltration of the algal cell. *Orciraptor* cells which infiltrated an algal cell fed internally by phagocytosis and were typically observed to reside inside during digestion of the food (Figure 2E,F). As known from its parasitoid relative *Viridiraptor invadens* (Hess & Melkonian, 2013), *Orciraptor* was also observed to perforate algal walls from the inside to leave the cell (Figure 2G). Larger filamentous species (*Spirogyra* sp.) were consumed by both partial protoplast extraction (Figure 2H) and infiltration (Figure 2I). In the algal filaments, *O. agilis* also perforated the cross walls (Figure 2J), but we never observed the extensive lateral propagation as documented for *V. invadens* (Hess & Melkonian, 2013). In addition, *Orciraptor* fed readily on released algal cell contents by simple phagocytosis (Figure 2K). There were few zygnematophyte strains that were resistant to *Orciraptor*, namely *Serritaenia* species and *Mesoteanium caldariorum*. These algae were attacked by *Orciraptor*, but after multiple hours of contact no perforations or feeding events were observed.

**FIGURE 3:**
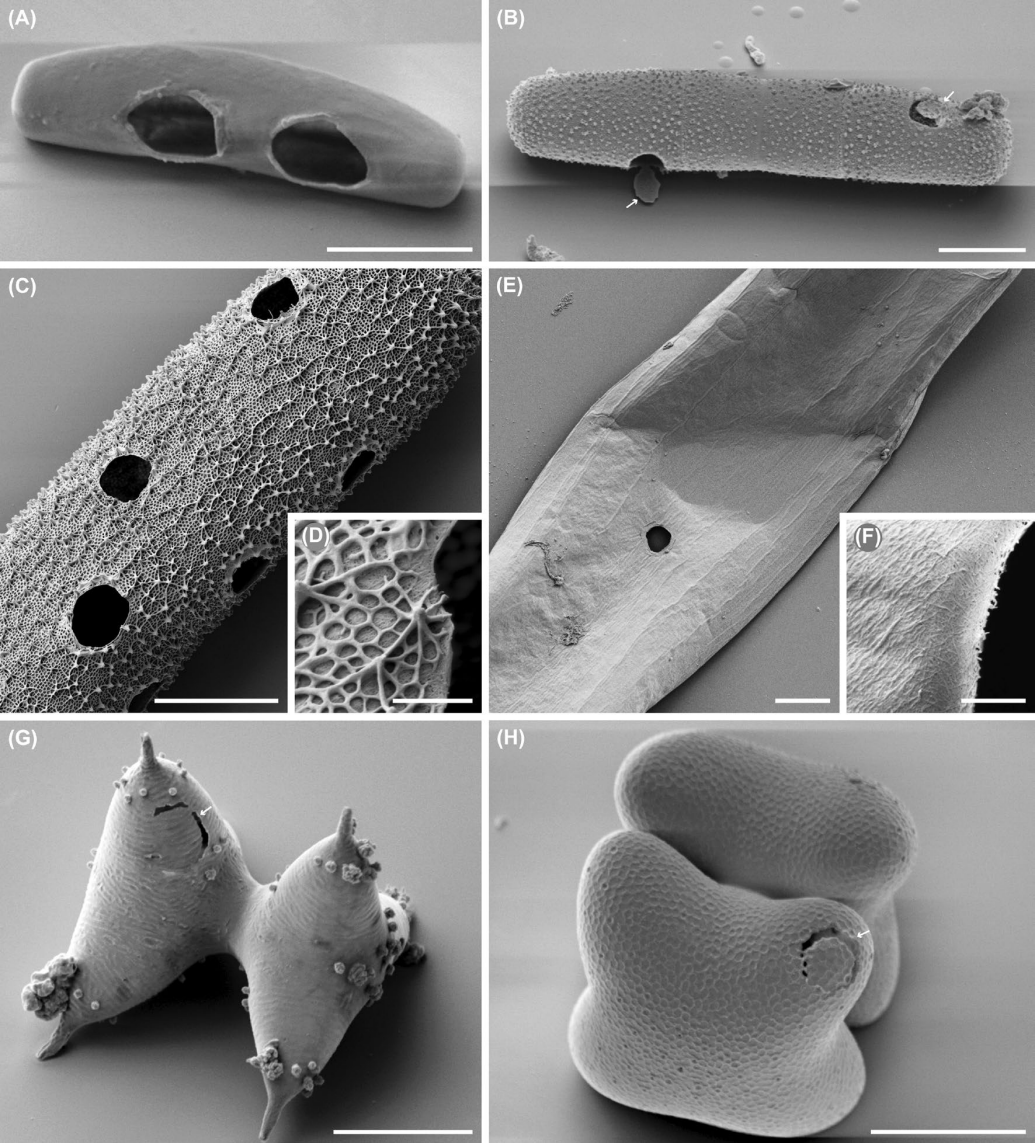
Scanning electron micrographs of zygnematophytes perforated by *O. agilis*. (A) *Roya obtusa*. (B) *Planotaenium ohtanii* with attached cell wall discs (arrows). (C) *Penium margaritaceum* with multiple perforations. (D) Close‐up of the reticulate cell wall of *Penium margaritaceum*. (E) *Planotaenium interruptum*. (F) Close‐up of the perforation in *Planotaenium interruptum*. (G) *Staurodesmus mamillatus* with incomplete perforation (arrow). (H) *Cosmarium tinctum* with circular perforation (arrow) and cell wall disc still in place. Scale bars: 10 μm.

SEM allowed a close look at the perforations in selected zygnematophyte strains. *Orciraptor* perforated algae with varying cell wall structure and thickness, e.g. the smooth‐walled *Roya obtusa* (Figure 3A) and the decorated *Planotaenium ohtanii* (Figure 3B). The relative number of cell wall discs attached to the emptied algal cells differed depending on the species but did not clearly correlate with the visible cell wall properties. In all cases a degradation of different cell wall layers, namely the amorphous pectic component (smooth or reticulate) and the cellulose microfibrils could be documented (Figure 3C–F). Algal strains which were attacked but did not support significant growth of *Orciraptor* often exhibited incomplete perforations, e.g. *Staurodesmus mammilatus* (Figure 3G) and *Cosmarium tinctum* (Figure 3H).

### Gelatinous colonies (Chlorophyceae and Cryptophyceae)

**FIGURE 4:**
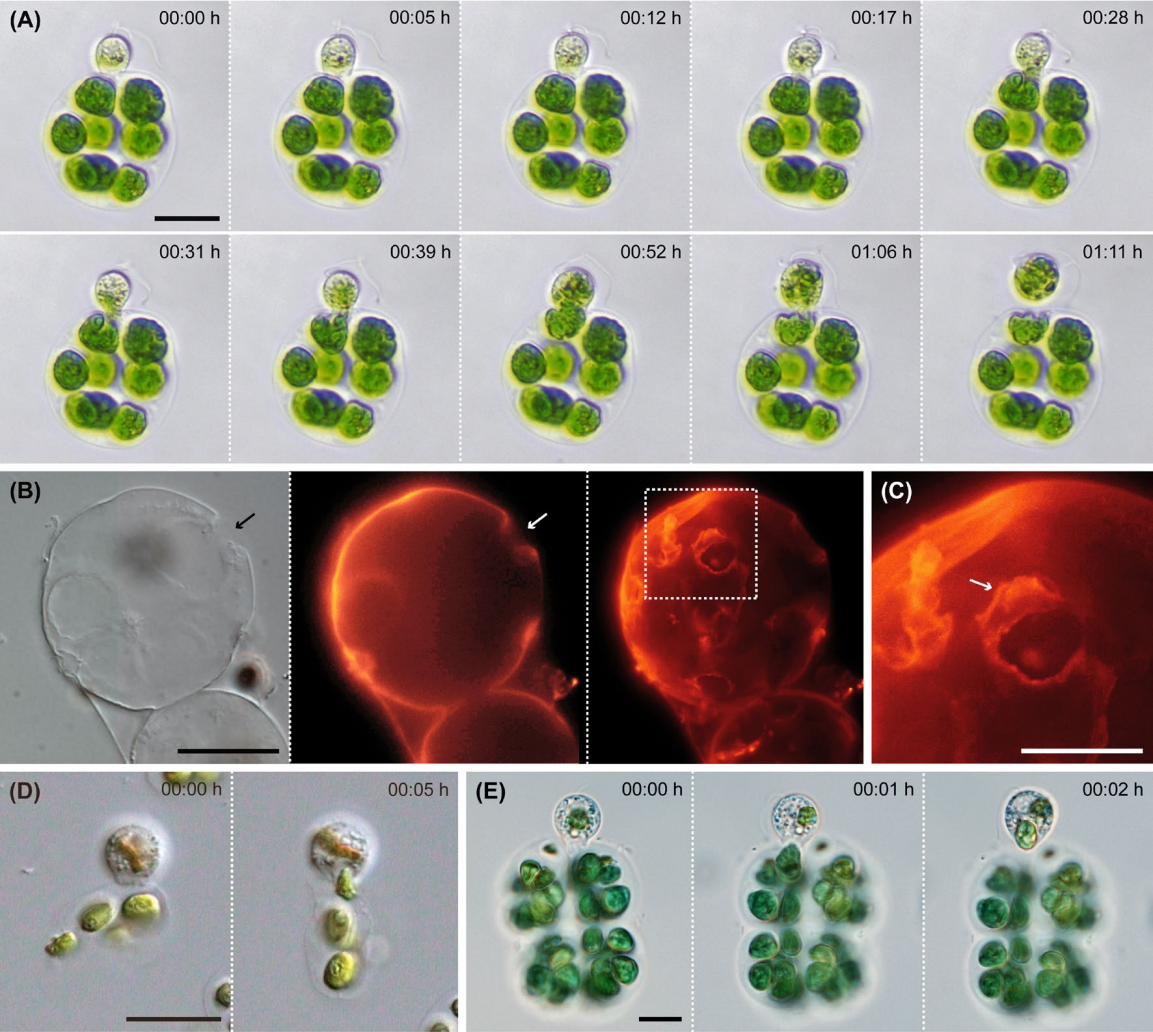
*Orciraptor agilis* feeding on gelatinous colonies and palmella. (A) Time series of *O. agilis* feeding on the cell contents of *E. elegans* after piercing the gelatinous colony. (B) Colony of *E. elegans* emptied by *O. agilis* with discernible degradation of the outer mucus layer (arrows). The staining with fluorescent wheat germ agglutinin shows the perforations and a flap of displaced mucus. (C) Close‐up of displaced mucus (arrow) from (B). (D) *O. agilis* removing a complete cell from *Pandorina morum*. (E) *O. agilis* removing a cell from a palmella of *Chroomonas* sp. Scale bars: A, B, D, E: 20 μm, C: 10 μm.

*Orciraptor* attacked gelatinous colonies of the Chlamydomonadales (Chlorophyceae) and palmella of *Chroomonas* sp. (Cryptophyceae). During the interaction with *E. elegans*, we observed the formation of a lysopodium and the lysis of the outer mucilage layer of the algal colony, followed by the perforation of individual algal cells. This process took about 30 min. Subsequently, *Orciraptor* extracted parts of the algal protoplast while the main cell body stayed outside the colony (Figure 4A; Video S1). The emptied colonies of *E. elegans* exhibited discrete perforations of 5–10 μm (*n* = 9), sometimes with a flap of gelatinous material. The latter could be visualized by fluorescence microscopy after staining with fluorescent wheat germ agglutinin (Figure 4B,C). Furthermore, we observed the removal of entire cells from the colonies of *Volvox tertius*, *Volvox aerius* and *Pandorina morum* by phagocytosis (Figure 4D). This was not preceded by a distinct perforation phase and rather resembled a typical phagocytotic uptake of free cells. *Orciraptor* acted in a similar manner on the palmella of the cryptophyte *Chroomonas*. Just within a few minutes, it removed *Chroomonas* cells from the mucilaginous palmella by pseudopodial action (Figure 4E; Video S2).

### Euglenid flagellates (Euglenophyceae)

**FIGURE 5:**
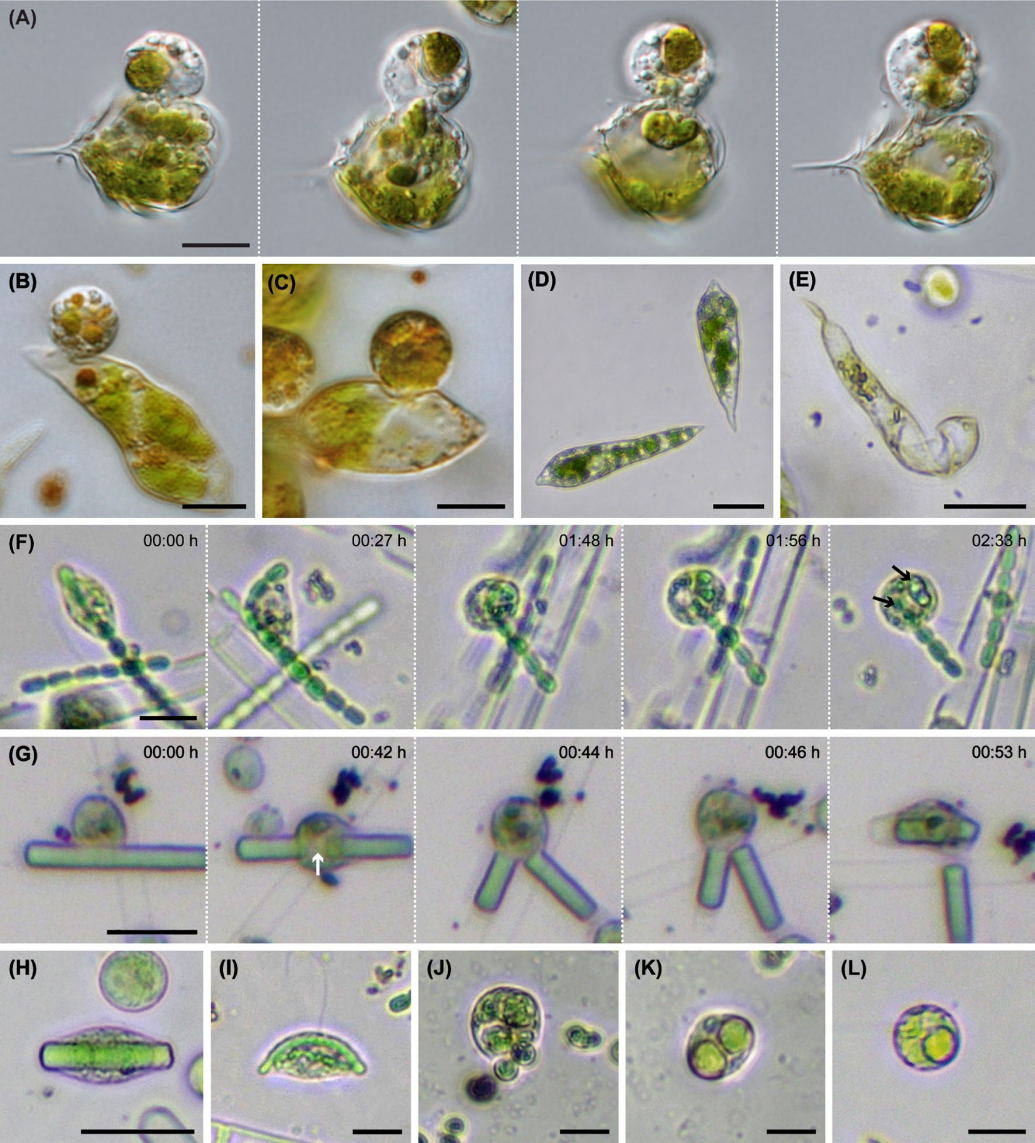
Interaction of *O. agilis* with euglenids, Cyanobacteria, yeast, and wheat flour. (A) *O. agilis* extracting the cell contents of *M. pseudonordstedtii*. (B, C) *O. agilis* extracting cell contents of *Euglena deses*. (D) Freeze‐killed cells of *Euglena deses*. (E) Remains of *Euglena deses* after extraction by *O. agilis*. (F) Time series of *O. agilis* incorporating and lysing a filament of an *Anabaena* species. Note the removed fragments (arrows). (G) Time series of *O. agilis* lysing and incorporating a filament of *Oscillatoria amoena*. (H, I) Cells of *O. agilis* deformed by filaments of *Oscillatoria amoena* (H) and *Anabaena* sp. (I). (J, K) *O. agilis* with incorporated cells of *Saccharomyces cerevisiae*. (L) *O. agilis* with incorporated amyloplast from wheat flour. Scale bars: 20 μm.

*Orciraptor* consumed all offered euglenids, including two metabolic *Euglena* species (*E. deses*, *E. mutabilis*) and the rigid *Monomorphina pseudonordstedtii*. It attached to various regions of the dead euglenid cells, formed a lysopodium, perforated the pellicle, and extracted the cell contents (Figure 5A–C). Euglenid cells were never invaded by *Orciraptor*. A comparison of *Euglena deses* cells before and after feeding revealed that *Orciraptor* crumbled the pellicle of the metabolic euglenids during the feeding process (Figure 5D,E).

### Bacterial phototrophs (Cyanobacteria)

*Orciraptor* fed on the dead cyanobacteria *Oscillatoria amoena* and *Anabaena* sp., but we frequently observed unsuccessful feeding attempts and the growth of the cultures appeared very slow. *Orciraptor* applied two different feeding strategies, both of which involved the lysis of cyanobacterial cells. Filaments which were ingested from one end but too long to be incorporated entirely were lysed internally (Figure 5F). This process took up to 1 h, while complete uptake of smaller filaments was achieved in just a few minutes. *Orciraptor* also lysed cyanobacterial cell walls externally, either laterally (from the side of the filament) or terminally, followed by the phagocytosis of the cell contents (Figure 5G). After the uptake of filaments or parts thereof, the *Orciraptor* cells appeared heavily deformed (Figure 5H,I).

### Hyphal fungi and yeasts (Letiomycetes, Saccharomycetes, and Tremellomycetes)

*Orciraptor* was offered several strains of aquatic hyphomycetes (genera *Articulospora*, *Amniculicola*, *Fontanospora*, *Lemonniera*, *Vibrissea*), but did not show any attacking attempts or interactions with the hyphae of the fungi. After a few days the *Orciraptor* cells died off. In contrast, dead yeast cells from different orders (Saccharomycetales, Tremellales, Filobasidiales) were phagocytosed entirely (Figure 5J,K). However, after processing (partial digestion?) no consecutive feeding was observed and the *Orciraptor* cells starved to death.

### Dead plant amyloplasts (wheat flour)

Parts of the wheat flour lumps were taken up by *Orciraptor* through usual phagocytosis (Figure 5L)—similar to the uptake of free cell contents of damaged algae (see above). Even though the *Orciraptor* culture was viable on flour, the culture grew very slowly and the *Orciraptor* cells stayed mainly small.

## DISCUSSION

In this study we tested the interaction of *O. agilis* with organisms of different cellular organization, morphology, and biochemistry in the live and dead condition. This revealed that *Orciraptor* can feed and proliferate on a variety of microbial organisms, including coccal and filamentous green algae, phototrophic flagellates, colonial green algae, and Cyanobacteria. However, these organisms were only accepted in the dead condition, supporting that *O. agilis* is a necrophage as previously suggested on the basis of natural observations and a limited feeding experiment (Hess & Melkonian, 2013). Interestingly, *O. agilis* showed very limited interaction with fungal cells, and no feeding on the tested aquatic hyphomycetes. The latter are typical inhabitants of standing waters, in which they decompose submerged leaf litter (Ingold, 1942; Tsui et al., 2016), and expected to co‐occur with *O. agilis* in the natural habitat (shallow bog ponds with submerged plant material).

*Orciraptor agilis* has the capacity to recognize and perforate dead cells and organisms with major differences in their surface properties. While the zygnematophycean green algae have plant like cell walls with cellulose and pectins as major components, the Chlamydomonadales are embedded in a glycoprotein‐rich mucilage (Coleman, 2012; Goodenough & Heuser, 1985). The euglenophytes do not bear a rigid cell wall, but a pellicle composed of proteinaceous intracellular strips (Leander, 2004), and the Cyanobacteria are bounded by gram‐negative bacterial walls with peptidoglycan (Jost, 1965; Stevens Jr. & Nierzwicki‐Bauer, 1991). The fact that *O. agilis* can penetrate algae of these very different taxonomic groups indicates that it must exhibit a rich toolkit of binding proteins and lytic enzymes, for example, carbohydrate‐active enzymes (CAZymes) and proteases. This aligns well with the surprising diversity of CAZymes previously detected by mRNA sequencing in this species (Gerbracht et al., 2022) and inspires for studying the modulation of CAZyme expression in response to various food sources. Furthermore, we observed major differences in the feeding success of *O. agilis* with different zygnematophyte species. The tested algal strains displayed cell walls of different thickness and microstructure, indicating that these cell wall properties determine the susceptibility of zygnematophytes to attack from other organisms. Notably, *O. agilis* did not accept the saccoderm demids of the Serritaeniales (*Serritaenia* spp.), which have relatively thin and smooth walls (Busch & Hess, 2022). These algae might have other deterring or masking traits (e.g. mucus sheaths), which are yet to be explored. It is still unknown how *O. agilis* recognizes suitable food organisms. However, the fact that it did not attack live organisms points to relatively universal, chemical compounds released from dead cells that might act as early‐stage cues.

Based on the observations in natural samples, *Orciraptor* was first described as a highly specialized protoplast feeder on dead zygnematophytes (Hess & Melkonian, 2013). This sophisticated feeding strategy includes the formation of an F‐actin‐rich, ring‐shaped contact zone (lysopodium), which probably works as a template for cell wall perforation (Busch & Hess, 2017), and the well‐defined secretion of an endocellulase directed against the major carbohydrate component of zygnematophycean walls (Moye et al., 2022). And yet, as we demonstrate here *Orciraptor* shows a stunning range of feeding strategies. Depending on the organization of the food item, it performs (1) protoplast extraction (zygnematophytes, euglenids, Chlamydomonadales), (2) infiltration (filamentous green algae, large coccal cells), (3) extraction of cells from gelatinous colonies (Chlamydomonadales, cryptophyte palmella), and (4) free capture (yeast cells, free protoplast material, flour). In addition, *O. agilis* can lyse partially ingested cyanobacteria and thereby “bite off” portions of filaments. Similar feeding habits on filamentous but live cyanobacteria were documented for nucleariid amoebae, e.g. *Nuclearia thermophila* and *N. delicatula* (Dirren et al., 2017), which, however, belong to the Opisthokonta and are thus phylogenetically very distant to *Orciraptor*. We are just at the beginning of exploring this trophic flexibility in protists, and their ability to consume several distinct organismal groups by different strategies seems to be underrated. Other examples of flexible, prey‐dependent changes in feeding strategy were so far found in the vampyrellid amoebae, e.g. in *Sericomyxa perlucida* (More et al., 2021), *Leptophrys vorax* and *Arachnomyxa cryptophaga* (Hess, 2017). It was shown on some testate amoebae that prey preferences of protists influence the placement of species in the microbial food web and the trophic levels (Jassey et al., 2012, 2013). This aspect is especially relevant for species with wide food ranges and flexible feeding behavior—trophic versatility might result in different ecological niches to be occupied.

Overall, the extent of necrophagy in protists and its importance in natural environments is still poorly explored. There are relatively few examples of documented (facultative) necrophagy, e.g. in ciliates and in testate and naked amoebae (Cann, 1986; Cienkowski, 1865; Fenchel, 1969; Han et al., 2011; Patterson, 1983). Here, we add some knowledge about a necrophagous nanoflagellate, which consumes various dead phototrophic microbes and potentially represents an obligatory broad‐range necrophytophage. Most heterotrophic nanoflagellates are thought to be associated with bacterivory and predation (Andersen & Fenchel, 1985; Boenigk & Arndt, 2002; Hehenberger et al., 2017; Howe et al., 2009; Tikhonenkov, 2020). In fact, the close relatives of Viridiraptorids (other glissomonads and pansomonads) are mostly soil‐dwelling species reported to feed on live bacteria and protists (Howe et al., 2009, 2011; Vickerman et al., 2005). It will be interesting to test these widespread cercozoan flagellates for necrophagy and to assess whether this is a common (but overlooked) aspect of the feeding ecology of nanoflagellates or a derived specialty of viridiraptorids. Furthermore, there are open questions about selective feeding in necrophagous viridiraptorids under natural conditions and potential population dynamics of such species in response to the accumulation of dead algal masses. It seems likely that *Orciraptor* is involved in the seasonal breakdown of dead indigenous algae in shallow moorland waters. Such necrophagous protists could potentially fulfill important roles in “cleaning up” the environment and releasing nutrients from dead biomass for the live microbiota.

## Supporting information


Video S1.

Video S2.

Data S1.
