# Supplementary material for: Broad‐range necrophytophagy in the flagellate Orciraptor agilis (Viridiraptoridae, Cercozoa) and the underappreciated role of scavenging among protists
Source: J Eukaryot Microbiol. 2024 Nov 3;72(2):e13065. doi: 10.1111/jeu.13065 (PMC11822879; doi:10.1111/jeu.13065)
Supplement: Supplementary file 3 — Data S1. [file JEU-72-e13065-s003.docx]

**Video caption**

**Video S1.** Orciraptor agilis extracting cell contents of a dead colony of Eudorina elegans. Time-lapse.

**Video S2**. Orciraptor agilis extracting cells of Chroomonas sp. by pseudopodial action. Time-lapse.
